# Inter-User Distance Estimation Based on a New Type of Fingerprint in Massive MIMO System for COVID-19 Contact Detection

**DOI:** 10.3390/s22166211

**Published:** 2022-08-18

**Authors:** Siyuan Yang, Mondher Bouazizi, Yuwen Cao, Tomoaki Ohtsuki

**Affiliations:** 1 Graduate School of Science and Technology, Keio University, Yokohama 223-8522, Japan; 2 Department of Information and Computer Science, Faculty of Science and Technology, Keio University, Yokohama 223-8522, Japan

**Keywords:** fingerprint, localization, 5G, super-resolution, deep learning

## Abstract

In this paper, we address the challenging task of estimating the distance between different users in a Millimeter Wave (mmWave) massive Multiple-Input Multiple-Output (mMIMO) system. The conventional Time of Arrival (ToA) and Angle of Arrival (AoA) based methods need users under the Line-of-Sight (LoS) scenario. Under the Non-LoS (NLoS) scenario, the fingerprint-based method can extract the fingerprint that includes the location information of users from the channel state information (CSI). However, high accuracy CSI estimation involves a huge overhead and high computational complexity. Thus, we design a new type of fingerprint generated by beam sweeping. In other words, we do not have to know the CSI to generate fingerprint. In general, each user can record the Received Signal Strength Indicator (RSSI) of the received beams by performing beam sweeping. Such measured RSSI values, formatted in a matrix, could be seen as beam energy image containing the angle and location information. However, we do not use the beam energy image as the fingerprint directly. Instead, we use the difference between two beam energy images as the fingerprint to train a Deep Neural Network (DNN) that learns the relationship between the fingerprints and the distance between these two users. Because the proposed fingerprint is rich in terms of the users’ location information, the DNN can easily learn the relationship between the difference between two beam energy images and the distance between those two users. We term it as the DNN-based inter-user distance (IUD) estimation method. Nonetheless, we investigate the possibility of using a super-resolution network to reduce the involved beam sweeping overhead. Using super-resolution to increase the resolution of low-resolution beam energy images obtained by the wide beam sweeping for IUD estimation can facilitate considerate improvement in accuracy performance. We evaluate the proposed DNN-based IUD estimation method by using original images of resolution 4 × 4, 8 × 8, and 16 × 16. Simulation results show that our method can achieve an average distance estimation error equal to 0.13 m for a coverage area of 60 × 30 m^2^. Moreover, our method outperforms the state-of-the-art IUD estimation methods that rely on users’ location information.

## 1. Introduction

With the advances and development in cellular network technology as well as artificial intelligence technology, several new apps have arisen, some of which are very reliant on the accuracy of the users’ position estimation [1,2]. It still is a challenge to find a robust solution to achieve the needed degree of precision in environments with rough multi-path channel conditions. The most common approach to localizing multipath channels relies on sensing technology that mitigates multipath effects [3] or fuses multiple sources of information [4].

More recently, with the COVID-19 pandemic, newer and stricter requirements have been in demand for applications built on the idea of identifying one’s exposure to other individuals carrying the virus. COVID-19 Contact Confirming Application (COCOA) is a kind of smartphone app that enables users to detect nearby people who were infected with the novel coronavirus. This app uses Bluetooth to connect with other users’ smartphones directly. If a user is infected, the COCOA in his smartphone will send the notifications to other users’ devices. However, this kind of Device-to-Device (D2D) communication needs the user to permit to allow other devices to connect to his/her device. If someone does not give such permission, he cannot send a COVID-19 alert notification to other users or receive the alert from others. In this sense, D2D communication alone is not enough. As such, an efficient localization method is needed. The base station (BS) can use the localization technique to know all users’ locations. When someone is infected, the BS can send notifications to nearby users. A more interesting task would be collocation identification. Collocation (also referred to as co-location) refers to the task of identifying users or groups of users within a certain range from one another and estimating the distance between them. This technology, up to very recently, relies on one of two main ideas that location detection techniques similar to the ones mentioned previously or exploiting the limited range wireless connections such as Bluetooth [5] and WiFi. However, the latter set of techniques (i.e., Bluetooth and WiFi-based techniques) may have some drawbacks [6], such as limited coverage, efficiency, and availability. Besides, there are some radar-based solutions for multi-user distance estimation [7,8]. M. Mercuri et al. [7] proposed a single-input and single-output (SISO) frequency-modulated continuous wave (FMCW) radar architecture. The radar sensor integrates two frequency scanning antennas. Their method demonstrates that it is possible to successfully locate the human volunteers, at different absolute distances and orientations. However, in the outdoor scenario, detecting the user distance under the interference of many signals, noise, and obstacles between the users and the sensors is a challenge. In that sense, the former family of approaches (i.e., the use of cellular network-based localization) has more promise. For instance, the use of uni-directional signals brings promise to improve the methods of cellular network-based localization, which could lead to a better co-location identification. Several studies in the last few decades proposed the use of cellular networks for outdoor localization. Despite the positive results obtained using 4G Long Term Evolution (LTE) networks [9], the nature of 4G signal propagation makes it difficult to develop a highly accurate positioning system. Millimeter Wave (MmWave) signals have a high temporal resolution due to their propagation characteristics, making precise positioning possible. Therefore, recent works have proposed exploiting the mmWave signal of the fifth-generation (5G) of mobile connectivity in applications related to positioning and localization [10,11,12,13,14,15,16,17,18,19,20,21,22,23,24,25,26].

The rise of 5G cellular networks shows that mmWave massive Multiple-Input Multiple-Output (mMIMO) provides exceptionally accurate localization [27,28]. In such a case, mmWave massive MIMO has attracted the attention of the research community and industry alike, for several reasons, including its high data rates, energy efficiency, and low latency [29]. In particular, a major advantage that motivated us to use mmWave massive MIMO for this paper is its high spatial resolution and beam-directing capability that can be used to pinpoint users very accurately [30]. While massive MIMO-OFDM systems are capable of high-resolution fingerprint extraction, creating a database including all locations/UEs fingerprints is a heavy burden due to their relatively high storage and matching overhead [31]. To solve these problems, deep neural networks (DNN) can extract features from the high-resolution fingerprints and match it to the users’ position without high storage and high matching overhead. In [28,32,33,34,35,36], the researchers have studied the application of DNN for user localization. All of these works use channel state information (CSI) as the fingerprint or need to extract the fingerprint from CSI. However, how to get the CSI with high accuracy and without high CSI estimation overhead is another problem that needs to be addressed. Localization techniques are also a good option for determining inter-user distances (IUDs). With the use of location techniques, users’ locations can be determined up to pinpoint accuracy, at which point the distances between them (i.e., the users) can be calculated. On the other hand, the disadvantage of these systems is that they depend on accurate location determination, which can be computationally expensive or perhaps requires more than one BS to operate properly [27,37]. The limitations stated above motivate us to find a novel method to determine the IUD with high accuracy in the communication environment with only one BS. The rest of the paper is organized as follows. Section II describes some of the related work and presents our motivations for this works. Section III explains the system model and the channel model. We present in Section IV and V the proposed method. The simulation results are shown and discussed in section VI. Finally, the Conclusion is shown in Section VII. The Key notations used in this article are listed in Table 1.

## 2. Related Work and Motivations

### 2.1. Related Work

In 5G mmWave networks, there are some technologies of user localization. The first kind of technique predicts the users’ location by using the estimated Time of Arrival (ToA) [37,38,39], Angle of Arrival (AoA) [11,27,40,41,42,43]. For instance, the authors in [11] achieved the localization error in the range from 0.16 m to 3.25 m by using data fusion and machine learning to estimate the ToA and AoA at users. In [37], Abu-Shaban et al. investigated 3D and 2D Line-of-Sight (LoS) localization situations and provided closed-form formulations for Position Error Bound (PEB) and Orientation Error Bound (OEB). The second kind of technique designs fingerprints using for localization [31,32,35,36,44,45,46,47,48,49,50]. In [35], Gante et al. developed a neural network architecture using the beamformed fingerprint for users localization. A short sequence of Convolutional Neural Networks (CNN) that achieves an average estimation inaccuracy of 1.78 m is proposed by them using for actual outdoor scenarios with primarily Non-Line-of-Sight (NLoS) locations. Savic et al. in [36] used received signal strengths as fingerprints for accurate users location estimation. Besides, they also gave a Gaussian process regression-based solution for fingerprint-based localization. Deep learning based technique was also studied for solving localization problem [28,32,33,34]. In [32], to improve the localization accuracy, they propose a Deep Convolutional Neural Network (DCNN) trained by Angle Delay Profiles (ADPs) as fingerprints.

When it comes to 5G mmWave cellular networks, IUD estimation has received little attention in the literature. This is most likely since it can be calculated very easily from a precise localization (if achieved). IUD estimation means detecting the distance between the users to obtain users’ locations whose in a certain range from one another. This IUD estimation technique can be used in wide kinds of scenarios. In the case of the COVID-19 pandemic, for example, it is feasible to determine who has been exposed to viral carriers over lengthy periods of time.

Bluetooth and WiFi are the most common technologies used for identifying users in proximity of one another [51,52,53,54,55,56,57,58]. However, such approaches require all the users to connect to the same WiFi hotspot or allow mutual Bluetooth connection to their devices so that location information is shared, thus identifying the exposure to the virus is possible. Besides, in the outdoor scenario, noise and other interference cause the position estimation accuracy cannot be very high. Thus, the goal of this study is to employ 5G mmWave networks instead, to estimate IUD more accurately by utilizing a unique fingerprint type. The BS can collect the potential virus carrier’s location information and notify other users nearby. We summarize the existing works in Table 2.

**Table 2:** The summary of the existing works.

Authors	Scenario	Method	Limitations
Mercuri et al. [7]	Indoors	A single-input and SISO FMCW radar architecture that integrates two frequency scanning antennas is proposed.	This method is valid only in the LoS scenario.
Kanhere et al. [11]	Indoors	Positioning using a combination of measured path loss and AoA.	The accuracy of positioning depends on the measurement accuracy.
Vieira et al. [32]	Outdoors	An ADP is obtained from the CSI and using the DCNN to learn the mapping between the ADP and the users’ location.	They extracted the ADP from the measured CSI. Thus, the accuracy of this method depends on the measurement accuracy.
Sun et al. [31]	Outdoors	An angle-delay channel amplitude matrix (ADCAM) is proposed as fingerprint. The ADCAM has rich multipath information with clear physical interpretation that can train DCNN easily.	They assume that the CSI is known.
Sun et al. [47]	Outdoors	A classification-based localization method is proposed.	This method need to know the CSI. Besides, this method needs a large storage overhead to save training data.

In the next section, we will go through this in more depth.

### 2.2. Motivations

In prior work [59], we introduced a unique fingerprint approach for predicting IUD. Our goal is to utilize a single BS to estimate the distance between each pair of distinct users, allowing us to identify users who were close to one another. By beam sweeping, we obtain the difference between the beam energy images of two users as fingerprints instead of using the CSI or ADPs in this study. Then, to estimate the distance between each pair of users, we offer a unique IUD estimation technique based on deep learning. To verify the robustness of our proposed method, we evaluate it after training in a new environment. We run the simulations on a new environment characterized by a different number and different locations of buildings. In the new environment, the trained model is expected to drop in the performance of IUD estimation. We analyze how much ground truth data are required to be collected for the trained model to be fine-tuned effectively in the new environment. We also study how many epochs the model is fine-tuned to achieve a distance estimation performance close to that of the model in the original environment. We identify the correlation between the fingerprint and the distance between distinct users in our technique, which reduces the estimation error significantly. Furthermore, because our technique does not rely on the individual fingerprints of individual users, but rather on how distinct they are from each other, it is more resilient to changes in the environment (for example, a change in BS). We also create a super-resolution neural network that can produce high-resolution beam energy pictures from low-resolution ones. The network is trained by feeding the network with actual down-sampled low-resolution images along with their real high-resolution counterparts. Through training, the network learns how to recreate the high-resolution images from low-resolution ones. The main contributions of this paper are summarized as follows:

- To improve the accuracy of the IUD estimation, we design a novel fingerprint that includes two users’ location information instead of using ADP or CSI as fingerprint which only includes the location information of one user.
- We propose a novel beam energy image generated by beam sweeping as the fingerprint. Compared with the conventional fingerprint-based methods, such as using the CSI or the extracted ADP from CSI as the fingerprint, the proposed fingerprint is deeply related to the horizontal and vertical angles corresponding to the user.
- Using beam energy image generated by beam sweeping instead of using CSI as a fingerprint can reduce the CSI estimation overhead.
- Compared with the conventional geometric methods which need multiple BSs for localization, the proposed method only needs one BS. Besides, after training, the proposed method can also work on mobile users.
- In general, generating a high-resolution beam energy image of a user by beam sweeping involves relatively high time expenditure. In this sense, we utilize a super-resolution technique to improve the low-resolution beam energy images to higher resolution ones.

## 3. System Model

### 3.1. Channel Model

**Figure 1:**
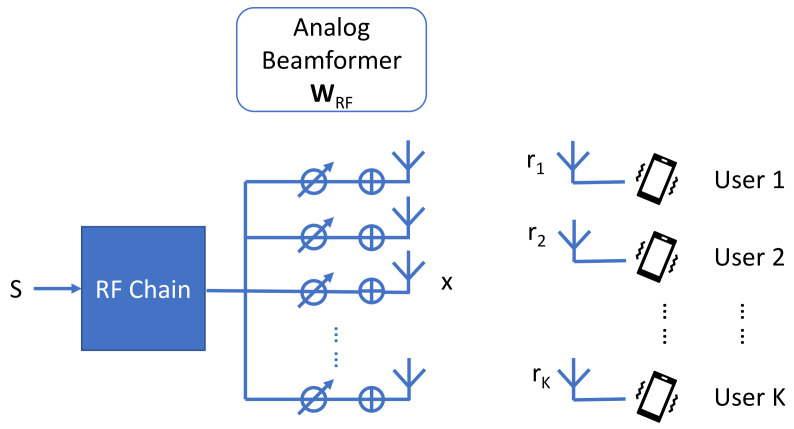
Illustration of a multi-user mmWave mMIMO system model.

In this paper, we consider a mmWave communication system with only one RF chain as shown in Figure 1 where one BS serves *K* users. In this system, the BS is equipped with a Uniform Planer Array (UPA) and the users are equipped, each, with a single antenna. The received signal of the *k*-th user $r_{k}$ is written as follows [55]:

(1) $$\begin{array}{cc} r_{k} & \;=\;\sqrt{P}\;h_{k}\;w_{RF}^{k}\;s_{k}\;+\;n_{k}, \end{array}$$

where *P* is the transmit power, $s_{k}$ is the transmitted signal of the *k*-th user, $w_{RF}^{k}\in W$ is the analog beamformer of the *k*-th user. Let W be the Discrete Fourier Transform (DFT)-based codebook for the UPA-based transmitter. W is used to apply an analog beamformer to create beams of the signal. $n_{k}∼CN\left(0,\sigma_{k}^{2}\right)$ is an additive white Gaussian noise (AWGN) with zero mean and variance $\sigma_{k}^{2}$ of the *k*-th user. Furthermore, the mmWave mMIMO channel $h_{k}\in C^{N_{t}}$ between the BS and the *k*-th user can be modeled by [55]:

(2) $$\begin{array}{cc} h_{k}\;=\; & \sqrt{\frac{N_{t}}{L}}\sum_{\ell \;=\;1}^{L}\alpha_{\ell }a\left(\varphi ,\phi \right), \end{array}$$

in which *L* denotes the total number of paths, φ and ϕ denote the horizontal and vertical angles, respectively. $\alpha_{\ell }$ denotes the complex path gain of the *ℓ*-th path. $N_{t}\;=\;N_{v}\;\times \;N_{h}$ corresponds to the number of transmit antennas at BS, here $N_{v}$ and $N_{h}$ denote the number of antennas along the vertical and horizontal, respectively. $a\left(\varphi ,\phi \right)\;=\;a^{v}\left(\phi \right)⊗a^{h}\left(\varphi ,\phi \right)$ denotes the steering vector, where $a^{v}\left(\phi \right)$ and $a^{h}\left(\varphi ,\phi \right)$ denote the steering vector over the horizontal axis and the vertical axis, respectively. Herein, $\alpha_{\ell }$ can be expressed as follows [60]:

(3) $$\alpha_{\ell }\;=\;\frac{\lambda \cdot g}{4\pi d_{p}^{\ell }}\sqrt{N_{t}}\cdot e^{\frac{\;-\;2j\pi d_{p}^{\ell }}{\lambda }},$$

where *g* is the complex reflection gain, $d_{p}$ is the path distance, and λ is the wavelength. The vertical and horizontal axes’ steering vectors can be expressed as follows [55]:

(4) $$a^{v}\left(\phi \right)\;=\;{\left[1,e^{\;-\;j2\pi d\cos(\phi )/\lambda },\cdots {,}^{\;-\;j2\pi \left(N_{v}\;-\;1\right)d\cos\left(\phi \right)/\lambda }\right]}^{T},$$

(5) $$\begin{array}{cc} a^{h}\left(\varphi ,\phi \right)\;=\; & [1,e^{\;-\;j2\pi d\sin(\phi )\sin(\varphi )/\lambda },\cdots ,\\ \; & e^{\;-\;j2\pi \left(N_{h}\;-\;1\right)d\sin\left(\phi \right)\sin\left(\varphi \right)/\lambda }{]}^{T}, \end{array}$$

where *d* is the distance between the consecutive antennas in both vertical and horizontal directions.

### 3.2. Beam Sweeping

**Figure 2:**
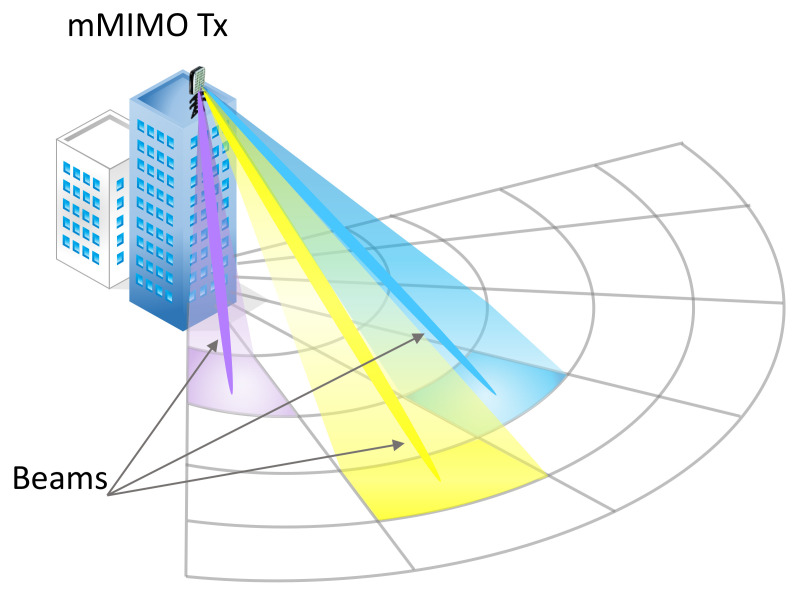
An example of beam sweeping to cover different regions in space.

Here we introduce a beam sweeping method based on the predefined beams in the codebook. In Figure 2, we divide the coverage into $N_{b}\;=\;n_{b}^{v}\;\times \;n_{b}^{h}$ sub-areas, where $n_{b}^{v}$ and $n_{b}^{h}$ denote the number of beams at the vertical axis and horizontal axis, respectively, and perform beam sweeping for different areas. When sweeping, unlike producing beams devoted to certain users, the beams are uniformly broadcasted to predetermined places rather than being directed to specific users. The set of the analog beamformers at the *n*-th region is represented by the matrix $W_{n}$ given as follows [55]:

(6) $$W_{n}\;=\;\left[w_{1,n}^{v}⊗w_{1,n}^{h},\cdots ,w_{K,n}^{v}⊗w_{K,n}^{h}\right],n\in \left\{1,\cdots ,N_{b}\right\},$$

where $w_{k,n}^{v}$ and $w_{k,n}^{h}$ are the weights on antenna elements along the vertical and horizontal directions, respectively. Our system assumes that the BS sweeps the generated beams first by broadcasting them in time slots. In other words, the BS broadcasts a signal with multiple beamformers to cover different locations for a certain number of time slots, as illustrated in Figure 2. The user measures the signals during the sweeping phase and sends the results to the BS through the mmWave control channel. We utilize the data to create a fingerprint that enables precise geographical location and co-location estimates in our research.

### 3.3. Problem Description

In the context of the system described above, we assume a number of *K* users that are within the coverage area of a specific BS. Throughout this work, we aim to achieve an objective that identifies the IUD. Similar to localization, the estimation of the distance between users relies on the beam energy images. However, it is achieved by comparing images of different users. We will demonstrate throughout this work that, despite being similar, our approach achieves much better performance in IUD estimation than user localization. In our system, the beam codebook is defined as the set of beams (also known as “codewords”) that are evenly disseminated in the downlink by the BS’s UPA. We assume that the UPA sweeps the beams in a common channel at consecutive times lots. Each user records the Received Signal Strength Indicator (RSSI) of the received beams as $m_{k}^{\left(x,y\right)}$ for $x\in \{0,\cdots ,N_{b}\}$ and $y\in \{0,\cdots ,N_{b}\}$ [43,50,61,62]. The measured RSSI values, formatted in a matrix, could be seen as an image, which we refer to as the beam energy image. Each image generated by a user is used as a fingerprint for its location which a neural network will use to estimate the location relative to the BS.

About our tasks, we show in this work the limitations of such a method in general in estimating the locations of the users, and its higher potential in estimating the distance between each pair of them. However, to briefly introduce the intuition behind it, we summarize in the following the main reasons. To begin with, the fingerprint of a given location is very dependent on the channel state and varies over time. Therefore, small changes in the channel and environment (e.g., level of noise, reflections, etc.) lead to inaccuracies in the location estimation. This change does not affect the IUD measurement, as the estimation is based on the difference between images generated at the same time rather than the images themselves. Therefore, instead of relying on location detection (which is a well-studied task) to measure the IUD, we aim at directly tackling this task (i.e., IUD estimation) using the differences in generated images by different users. The distance estimation problem could be expressed as a non-linear function of fingerprint images. Similarly, the distance between users might be represented as a non-linear and non-transitive function of the difference in the beam energy images created by various users. In other words, we formulate the problem as a mapping between the difference in beam energy images and the distance between any two users.

## 4. The IUD Estimation Approach

### 4.1. User Localization Based IUD Estimation

**Figure 3:**
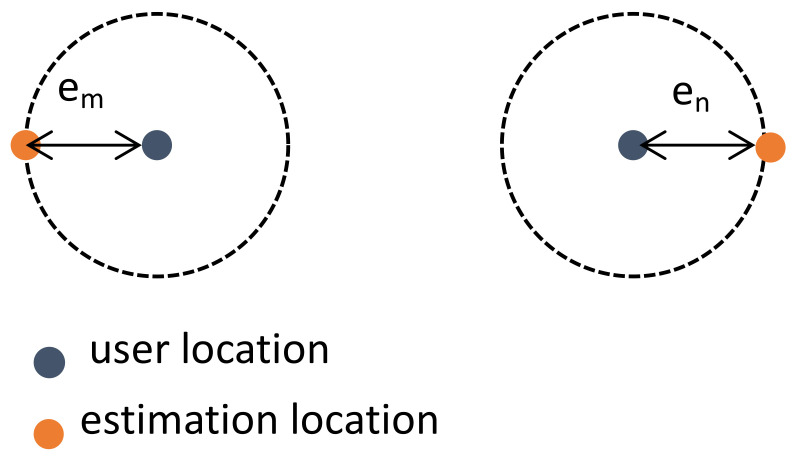
IUD estimation error of the conventional localization-based method.

Conventionally, to estimate the distance between two users, we need to first predict the location of the two users. Following are some works using the fingerprint-based method for user localization [31,32]. Vieira et al. [32] proposed a fingerprint-based user localization method. They used the measured channel snapshots of each user as fingerprints and trained the DCNN to predict users’ location in the massive MIMO system. Based on the DCNN-based method [32], Sun et al. [31] proposed a new type of fingerprint which they used for user localization in the massive MIMO system. Different from [32] which used the channel snapshots represented in the sparse domain as fingerprint, they proposed a fingerprint extraction method to extract the ADP from CSI as the fingerprint. Besides, they also propose a fingerprint compression method and clustering algorithm to reduce storage overhead and matching complexity. For the user localization problem, these methods can achieve high accuracy in estimating users’ location. As shown in Figure 3, we suppose that the estimation error of the user *m* and the user *n* are $e_{m}$ and $e_{n}$, respectively. Thus, the IUD estimation error $0\leq e\leq e_{m}\;+\;e_{n}$. However, if we can train a DNN for IUD estimation with the estimation error $e_{m}$ or $e_{n}$, the estimation error can go up to $\max\left(e_{m},e_{n}\right)\leq \left(e_{m}\;+\;e_{n}\right)$. Obviously, using user localization for IUD estimation is not the optimal solution.

### 4.2. Proposed IUD Estimations

**Figure 4:**
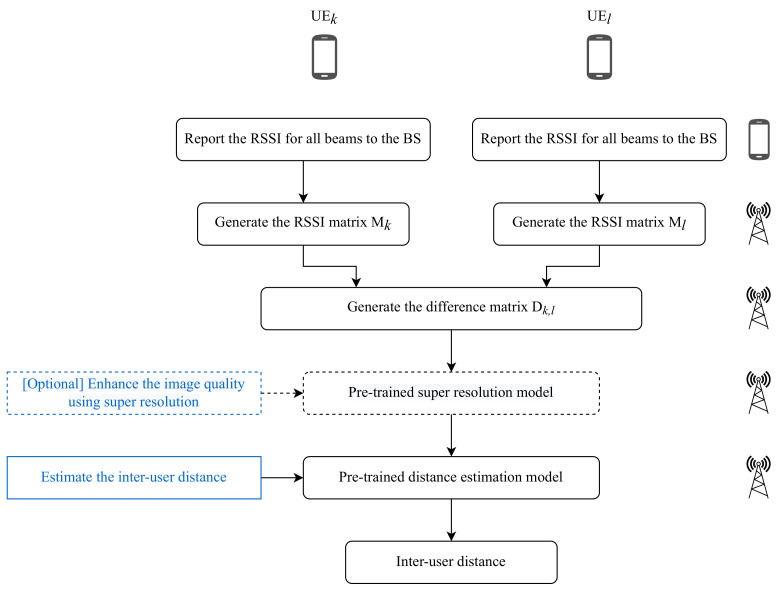
A flowchart of the proposed method for inter-user distance estimation.

For the above reasons, to improve the estimation accuracy of the distance between each pair of users, we develop a CNN to estimate the distance directly. In Figure 4, we show a flowchart of the overall proposed method. As shown in the flowchart, given two users *k* and *l*, these users report the RSSI for the received beams. The BS then uses the reported RSSI values to generate two images, one for each user, and then measures the difference between them as we will describe below. Using models trained offline, the image is either enhanced or used as it is to do a non-linear regression allowing for measuring the distance between *k* and *l*. We use the beam energy difference image of each pair of users as input instead of the beam energy image of each user. The output of the proposed CNN is the estimated distance between each pair of users. Given two users *k* and *l*, we denote by $M_{k}$ and $M_{l}$ their respective generated power matrices/images.

(7) $$M_{k}\;=\;\left[m_{k}^{\left(x,y\right)}\right],$$

where $x,\in \{1,\cdots n_{b}^{h}\}$ and $y,\in \{1,\cdots n_{b}^{v}\}$. As stated in the previous section, we refer to the number of beams as $N_{b}$ and we denote $n_{b}\;=\;\sqrt{N_{b}}$. We define the difference matrix between the two users *k* and *l* as:

(8) $$D_{k,l}\;=\;\left[\begin{array}{ccc} |m_{k}^{\left(1,1\right)}\;-\;m_{l}^{\left(1,1\right)}| & \cdots & |m_{k}^{\left(1,n_{b}\right)}\;-\;m_{l}^{\left(1,n_{b}\right)}|\\ |m_{k}^{\left(2,1\right)}\;-\;m_{l}^{\left(2,1\right)}| & \cdots & |m_{k}^{\left(2,n_{b}\right)}\;-\;m_{l}^{\left(2,n_{b}\right)}|\\ \vdots & \ddots & \vdots \\ |m_{k}^{\left(n_{b},1\right)}\;-\;m_{l}^{\left(n_{b},1\right)}| & \cdots & |m_{k}^{\left(n_{b},n_{b}\right)}\;-\;m_{l}^{\left(n_{b},n_{b}\right)}| \end{array}\right].$$

Hereafter, we will employ a much simpler notation for the above matrix:

(9) $$D_{k,l}\;=\;\left|M_{k}\;-\;M_{l}\right|.$$

**Figure 5:**
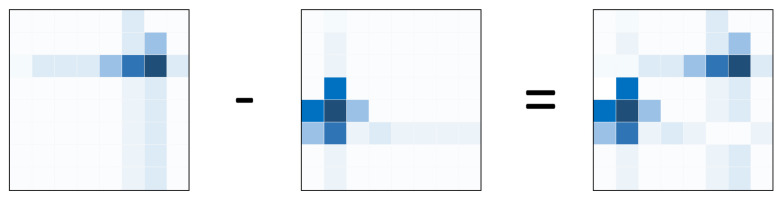
An example of visualization of the difference matrix.

In Figure 5, we offer an example of difference matrix visualization. The resultant matrix (the rightmost one) is sent into the neural network, which calculates the distance between the two users whose RSSI matrices are given in the leftmost part of the figure.

**Figure 6:**
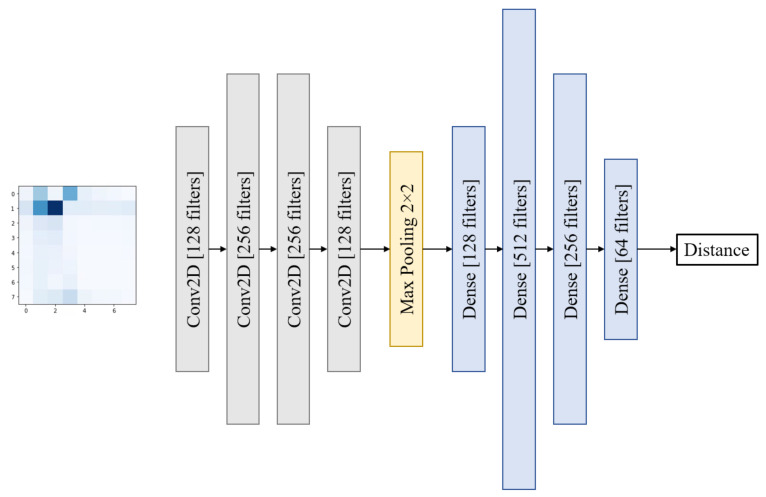
The architecture of the neural network used for distance estimation.

Figure 6 shows the neural network we used for distance estimation: As previously indicated, the input is the matrix $D_{k,l}$ representing the difference between the RSSI of the *k*-th user and *l*-th user for the different beams of a given granularity level. The neural network is made up of four convolution layers, each with a filter size of three times three, and a max-pooling layer with a size of 2 × 2. 128 filters, 256 filters, 256 filters, and 128 filters are used in the convolution layers, correspondingly. The max-pooling layer is followed by four completely linked layers of 128, 512, 256, and 64 neurons, respectively. Rectified Linear Unit is the activation mode for all of the aforementioned layers (ReLU). The network’s last layer is a dense layer with a single neuron and linear activation. This is since this neuron’s job is to assess the distance between users. The MSE between the actual distance between users (ground truth) and the anticipated distance (prediction) is used to train the network as the loss function that the network is designed to reduce. Here, given a batch *b*, with a batch size $S_{b}$, the loss function of the neural network is defined as:

(10) $$\mathrm{MSE}\left(X^{\left(b\right)},y^{\left(b\right)},F\right)\;=\;\frac{1}{S_{b}}\sum_{i\;=\;1}^{S_{b}}\left|\right|{\hat{y}}_{i}^{\left(b\right)}\left(F\right)\;-\;y_{i}^{\left(b\right)}{\left|\right|}^{2},$$

where $X^{\left(b\right)}$ is the set of the difference matrix $D_{k,l}$, $y^{\left(b\right)}$ is the ground truth distance between each pair of users, and ${\hat{y}}_{i}^{\left(b\right)}\left(F\right)$ is the estimated distance between the users by the proposed DNN network, where *F* denotes the weights of each layer of the proposed network. The above-mentioned network is designed to contain as few layers and parameters as feasible while yet working adequately. The performance of shallower networks suffers noticeably (MSE of the estimated distance), while the performance of deeper networks does not significantly increase over the suggested architecture. Because the network’s technological implementation (using Keras) necessitates defining the input shape (4 × 4 or 8 × 8), multiple networks were created. However, for clarity, we will refer to all of these network instances as if they were one.

### 4.3. Super-Resolution

**Figure 7:**
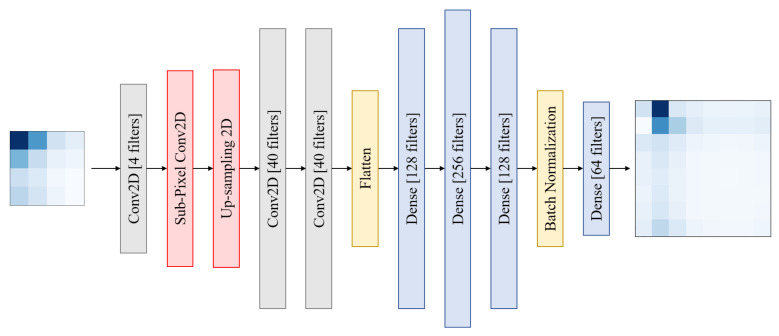
Architecture of the super-resolution neural network used.

As previously stated, the received power from uniformly distributed broad beams may be seen as pictures of various resolutions: 4 × 4 wide-beam received powers can be regarded as low-resolution 4 × 4 images, and 8 × 8 narrow-beam received powers can be regarded as higher resolution 8 × 8 images. The goal of super-resolution is to recover (or produce) high-resolution pictures from low-resolution ones in general. When applied to our approach, the ability to produce correct 8 × 8 beam images from 4 × 4 beam images enables the use of narrow beams to yield accurate fingerprints with co-localization accuracy comparable to that of broad beams. In our research, we used deep learning to implement a supervised approach to super-resolution. It’s worth noting that we experimented with many neural network designs throughout our early trials. However, because the outcomes of these networks were so similar, we will focus on the network that produced the greatest results, which is shown in Figure 7. The neural network consists of one convolution layer with four filters, a Sub-pixel convolution layer, and an up-sampling layer, which is followed by two convolution layers with 40 filters each. The output of the second layer is flattened and linked to three dense layers of 128, 256, and 128 neurons in succession. All of the layers above have Rectified Linear Unit as their activation (ReLU). After that, batch normalization is applied, followed by a dense layer with a linear activation and a number of filters equal to the predicted output’s number of pixels. The picture is supposed to be upscaled to 8 × 8 for 4 × 4 input photos. As a result, the number of neurons in the last dense layer is set to 64.

Because we’re employing a supervised technique, we’ll need a training set to teach the network how to build high-resolution pictures from low-resolution ones. To train the network, we employ the Mean Squared Error (MSE) as a loss function. Given a batch *b* with a size $S_{b}$, the MSE is defined as follows:

(11) $$\mathrm{MSE}\left(X^{\left(b\right)},y^{\left(b\right)},\theta \right)\;=\;\frac{1}{S_{b}}\sum_{i\;=\;1}^{S_{b}}\left|\right|{\hat{y}}_{i}^{\left(b\right)}\left(\theta \right)\;-\;y_{i}^{\left(b\right)}{\left|\right|}^{2},$$

$y_{i}^{\left(b\right)}$ and ${\hat{y}}_{i}^{\left(b\right)}\left(\theta \right)$ represent the ground truth high resolution beam-quality image and the image rebuilt using the network function θ, respectively.

## 5. Performance Evaluation and Simulation Results

### 5.1. Experiment Specifications

We run our experiments with collected data using Wireless Insite [63]. The channel model has been introduced in the Section 3. We generate 2 groups of datasets with different environments. The part of the first group is used for training, and the remainder of the first group and the second group are used for testing. The simulation parameters’ specifications are shown in Table 3. Keras and TensorFlow are used in all of our neural network implementations (for non-standard layers). To find suitable hyperparameters for the proposed DNN and super-resolution networks, we first try a learning rate of 0.1, the number of epochs of 10,000, and a batch size of 64. Upon obtaining a rough idea about the estimated accuracy and training time, we decided to use a smaller learning rate (i.e., a learning rate equal to 0.001), a smaller number of epochs (i.e., 1000 epochs), and a smaller batch size (i.e., a batch size equal to 32).

### 5.2. Evaluation Metrics

Throughout the rest of this section, we will be using different metrics to evaluate our proposed approach. Therefore, we define and explain here each of these metrics.

#### 5.2.1. Super Resolution

To evaluate the super-resolution neural network, we use the same loss function which we defined in Equation (11) which refers to an average of the MSE between the predicted pixel values and their real values. We show the average loss per epoch, which reflects how far the generated image is from the ground truth at each epoch.

#### 5.2.2. IUD Estimation

We evaluate our approach for IUD estimation by estimating the error between the actual distance and the one reported by the neural network. In other words, given two users *k* and *l*, we refer to the real distance between them as d(k,l), and the distance estimated by the neural network as $\hat{d}\left(k,l\right)$. The estimation error of the distance between the 2 users $e_{\mathrm{dist}}\left(k,l\right)$ is:

(12) $$e_{\mathrm{dist}}\left(k,l\right)\;=\;\sqrt{{\left(d\left(k,l\right)\;-\;\hat{d}\left(k,l\right)\right)}^{2}}.$$

Again, for visualization, we plot the CDF of $e_{\mathrm{dist}}$.

### 5.3. Super-Resolution Training

**Figure 8:**
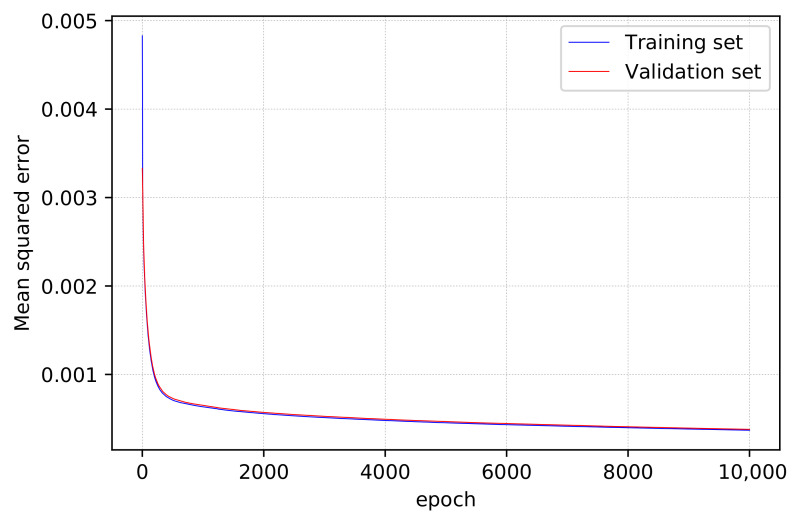
Training and validation loss during the training phase of the super-resolution neural network.

The loss in terms of MSE during the training phase of the super-resolution neural network is depicted in Figure 8. The neural network was still converging after 10 K epochs of training, and no over-fitting was observed: both the training and validation loss reduced constantly, indicating that training the network for more epochs can lead to higher performance. However, we stopped training at 10 K epochs and determined that the network had converged sufficiently for its output useful for our works. On the training set, the loss was $3.7\times 10^{\;-\;4}$, and on the validation set, it was $3.8\times 10^{\;-\;4}$. We will compare the results of distance estimation with and without super-resolution in the following sub-section to emphasize and genuinely assess the effectiveness of such procedures.

However, as previously noted, various super-resolution neural network algorithms have been explored, and the results are not far behind. This means that, regardless of the technology utilized to accomplish super-resolution, one can expect a performance improvement when compared to low-resolution photos.

### 5.4. Distance Estimation

**Figure 9:**
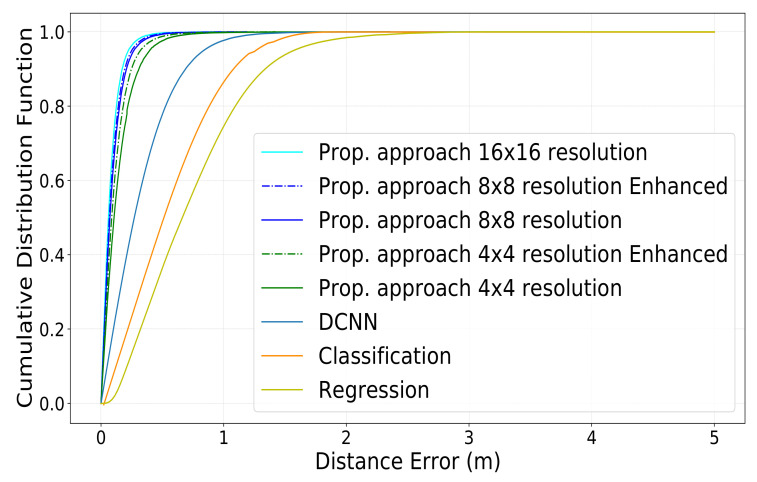
CDF of the distance estimation error for different transmit antennas configurations.

**Figure 10:**
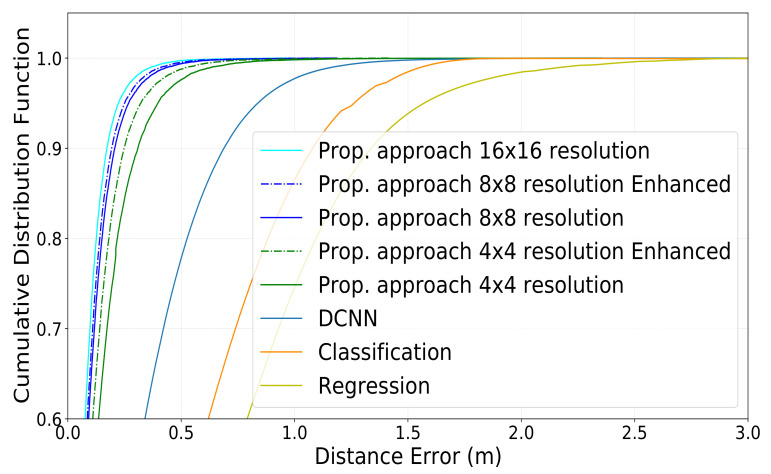
A zoom on the CDF of the distance estimation error for different transmit antennas configurations between CDF = 0.6 and CDF = 1.

The results of CDF of the distance error utilizing the different approaches we presented are shown in Figure 9 and Figure 10. The CDF of the distance error while utilizing 4 × 4 photos is shown in green. The consequences of adding super-resolution to the original 4 × 4 photos to upscale them to 8 × 8 are shown in particular by a dotted line. The CDF of the distance error when utilizing 8 × 8 pictures is shown in light blue. The consequences of adding super-resolution to the original 8 × 8 photos to upscale them to 16 × 16 are shown in particular by a dotted line. The CDF of the distance error while utilizing 16 × 16 pictures is shown in blue. We compared the proposed method with the DCNN location estimation method [32], the Regression-based method [47], and the Classification-based method [31]. The simulation results show that the proposed method has a significant improvement in the reduction of the IUD estimation error. As mentioned before, refs. [31,32,47] extract the ADP from CSI as the fingerprint. However, we use beam scanning to generate beam energy images that contain more angular and positional information than ADP. Furthermore, we use the difference between the beam energy images of the two users as the fingerprint, which simultaneously contains the location information of the two users. Thus, our proposed method achieves a better inter-distance estimation performance than the other methods.

In Table 4, we summarize the reported values of the error at CDF = 0.5 and CDF = 0.9 for ease of comparison. As we can observe, our proposed method outperforms the conventional ones [31,32,47], even when using wide beams (i.e., 4 × 4 ones).

At CDF = 0.5, our proposed approach reaches an error equal to 0.093 m for images of size 16 × 16, equal to 0.097 m for images of size 8 × 8, and equal to 0.160 m for images of size 4 × 4. More interestingly, when we employ super-resolution to upscale the 8 × 8 and 4 × 4 images to 16 × 16 and 8 × 8, our proposed method reaches an error equal to 0.096 m and 0.101 m, respectively, at CDF = 0.5. On the other hand, at CDF = 0.5, the best performance of the three conventional methods reaches an error equal to 0.280 m.

Similarly, when measuring the error at CDF = 0.9, our proposed approach reaches an error equal to 0.184 m for images of size 16 × 16, 0.205 m for images of size 8 × 8, and 0.304 m for images of size 4 × 4, respectively. After applying super-resolution to images of size 8 × 8 and images of size 4 × 4, the error decreases down to 0.231 m and 0.197 m, respectively. On the other hand, at CDF = 0.9, the best performance of the three conventional methods reaches an error equal to 0.703 m at best.

As can be shown, despite falling behind when conducting location detection, the proposed technique surpasses the standard one [31,32,47] in IUD estimates. This is because, unlike the traditional method, which maps fingerprints to location, our system learns to recognize the distance between users independent of where they are. Using such uncertain data twice for the inter-user estimate, however, increases the estimation error when measuring the position of individual users. It’s also worth noting that our strategy surpasses the traditional one in another way: the traditional method trains on exactly *N* occurrences given a set of *N* users in the training set, necessitating a high number of users for correct training. Our solution, on the other hand, will create a total of $\frac{N\cdot (N\;-\;1)}{2}$ instances for training for the *N* users, necessitating the simulation tool to generate data for fewer users to train the neural network effectively. We can observe that the narrower the beams are, the higher the detection accuracy is with our proposed technique. However, a more significant trend that we can see is that when the super-resolution approach is utilized, the results are considerably improved. This demonstrates the value of this method in terms of not only enhancing image quality but also refining distance estimates. Another advantage of our proposed strategy is that it is less likely to suffer performance degradation as a result of BS relocation. This is since it does not rely on the fingerprints themselves, but rather on the differences between them for various users. However, our strategy necessitates the training of two distinct networks: one for super-resolution and the other for distance calculation. With regards to the use of the highest resolution (i.e., 16 × 16) in particular, we can notice that, despite the improvement observed, this improvement is, in some sense, not justifiable: the power consumption and the time required to perform the beam sweeping are 4 times greater than when performing 8 × 8 sweeping, and 16 times greater than when performing 4 × 4 sweeping. Nonetheless, a similar increase in computation complexity is seen when training the neural network and inferring it. Similarly, applying super-resolution to 4 × 4 images has led to an improvement of over 50% and 31% in the error estimation at CDF = 0.5 and CDF = 0.9, respectively. However, after applying super-resolution on 8 × 8 images, the improvement reaches only 1% and 4% in the error estimation at CDF = 0.5 and CDF = 0.9, respectively, leading us to believe that the computation cost might not be justifiable in this case.

### 5.5. Robustness Analysis

To verify the robustness of our proposed method, we evaluate it in a new environment. Here, we run our simulations on an environment with different building structures on Wireless Insite [63]. The difference in buildings locations and orientations leads to different reflections, thus the trained model is expected to drop in performance of IUD estimation. However, rather than training the entire network from scratch, we fine-tune the already built model (i.e., the one created in the first environment) on the new environment using a limited number of users dispersed over its area, and using a limited number of epochs. In other words, the objective of this subsection is to estimate how much ground truth data are required, and how much should the model be re-trained to provide performance close to the original one.

#### 5.5.1. Fine-Tuning with Different Number of Users

As described above, since the model needs to be adjusted to fit the new environment, we need to fine-tune it with some data collected from this new environment. However, it is impractical to use the same number of users we used to first train the model. We need to evaluate how many user locations are needed to perfectly fine-tune the model. Given the different number of users (referred to as $N_{\mathrm{user}}^{i}$ where i∈{20,50,100}) whose location is known, we fine-tune the model using these data, and evaluate it on the entire region. The model is fine-tuned for 100 epochs using these data.

**Figure 11:**
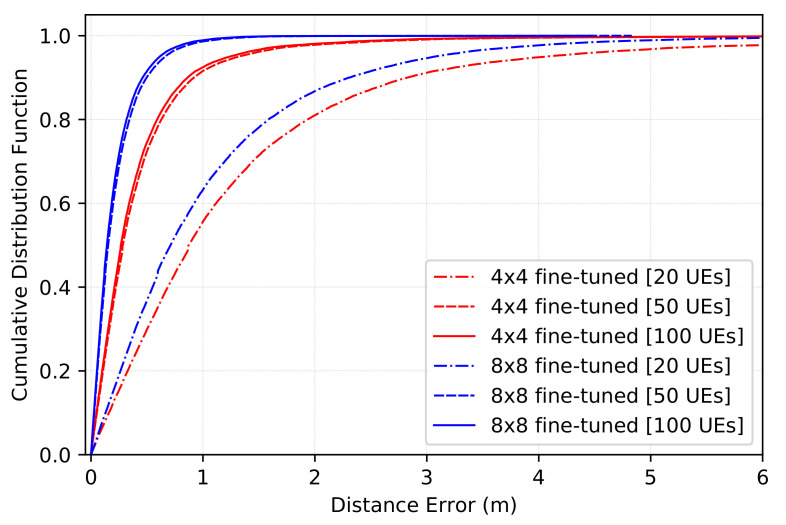
CDF of the distance estimation error on the new environment after fine-tuning the model for 50 epochs with 20, 50, and 100 users.

The CDF of the distance estimation error on the new environment for $N_{\mathrm{user}}^{20}$, $N_{\mathrm{user}}^{50}$, and $N_{\mathrm{user}}^{100}$ is given in Figure 11. Here, the red color refers to the fine-tuning of the model built for 4 × 4 images, and there blue one refers to the fine-tuning of the model built for 8 × 8 images. Nevertheless, the values at CDF = 0.5 and CDF = 0.9 are given in Table 5. As can be seen, after training the model with only 50 users, we reach a decent localization precision. For 4 × 4 images, the error reaches 0.284 m at CDF = 0.5, and 0.919 m at CDF = 0.9. This is not very far from the precision when using 100 users ’ data, where the error reaches for the same values of CDF 0.266 m and 0.862 m, respectively. The same behavior could be observed for images of size 8 × 8. When fine-tuning the network using 50 users, the error at CDF = 0.5 is equal to 0.153 m, and that at CDF = 0.9 is equal to 0.497 m for images of size 4 × 4. When using images of size 8 × 8, the error is equal to 0.142 m and 0.461 m, respectively.

#### 5.5.2. Fine-Tuning with Different Number of Epochs

Here again, we need to identify the minimum number of epochs required to fine-tune the model well enough to perform as well as the model in the original environment. Since we have concluded from the previous set of experiments that 50 users is enough to fine-tune the model efficiently, we use this same number in our next set of experiments. Here, we try the different number of epochs of training of the model to perform the overfitting. We refer to the number of epochs as $N_{\mathrm{epoch}}^{i}$ where i∈{10,50,100}.

**Figure 12:**
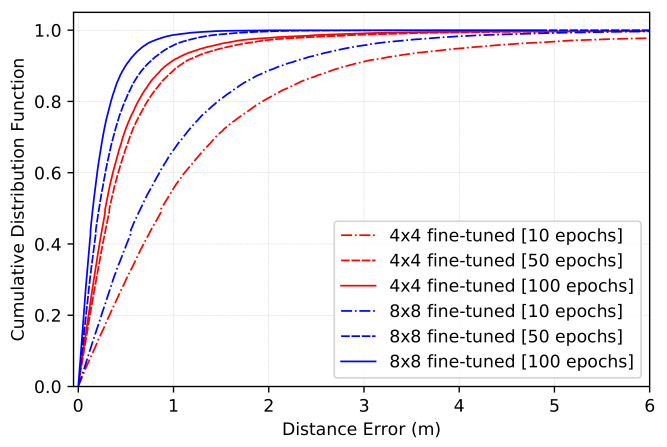
CDF of the distance estimation error on the new environment after fine-tuning the model for 10, 50, and 100 epochs.

The CDF of the distance estimation error on the new environment for $N_{\mathrm{epochs}}^{10}$, $N_{\mathrm{epochs}}^{50}$, and $N_{\mathrm{epochs}}^{100}$ is given in Figure 12. Here, the red color refers to the CDF of the fine-tuning of the model built for 4 × 4 images, and the blue one refers to the CDF of the fine-tuning of the model built for 8 × 8 images. In addition, the values at CDF = 0.5 and CDF = 0.9 are given in Table 6. Here, we can observe that after training the model for 10 epochs, the performance of the model is very poor, both when using 4 × 4 images and 8 × 8 images. In the case of 4 × 4 images, the error reaches 0.873 m at CDF = 0.5, and 2.826 m at CDF = 0.9. In the case of 8 × 8 images, the error reaches 0.655 m at CDF = 0.5, and 2.129 m at CDF = 0.9.

The precision improves when training for more epochs. For instance, after training the model for 50 epochs, and using images of size 4 × 4, the error at CDF = 0.5 and CDF = 0.9 reach 0.327 m and 1.060 m, respectively. Using images of size 8 × 8, these values reach 0.218 m and 0.710 m, respectively. When using 100 epochs, the precision improves even further: Using images of size 4 × 4, the error at CDF = 0.5 and CDF = 0.9 reach 0.284 m and 0.919 m, respectively. Using images of size 8 × 8, these values reach 0.153 m and 0.497 m, respectively.

### 5.6. Complexity Analysis

To estimate the complexity of our proposed method, we use the total number of parameters of the neural networks as an indicator. We have a set of convolutions, a set of dense layers, and a single max pooling layer. To that, we add the number of ReLU parameters. To recall, every convolutional layer is followed by a ReLU layer. The total number of parameters *P* of a given convolutional layer *c* is given by:

(13) P(c) = ((m·n·p) + 1)·k

where m and *n* are the width and height of each filter (3 × 3 in our case), *p* is the number of channels and *k* is the number of filters in the layer. The total number of parameters *P* of a given ReLU layer *a* is given by:

(14) P(a) = h·w·k

where *h* and *w* are the height and width of the input image, respectively, and *k* is again the number of filters. In addition to Equations (13) and (14), we use the following equation to calculate the total number of parameters *P* of a given dense layer *d*:

(15) P(d) = (s·t) + 1

where *s* is the size of the dense layer (the number of neurons) and *t* is the number of neurons in the previous layer.

As shown in Table 7, compared to the conventional methods, our neural network has a larger number of parameters than that of the other three methods. The total number of parameters of the classification neural network, when using input images of size 4 × 4, is about 1.4 M. When using input images of size 8 × 8, it is equal to 1.65 M, and when using input images of size 16 × 16, it is equal to 2.44 M. When applying the super-resolution technique, another network is being used, leading to a total number of parameters of about 2.86 M and 3.06 M for input images of size 4 × 4 and 8 × 8, respectively.

Compared to conventional methods that use shallow networks such as DCNN [32], our method might seem much more complex. However, it is important to keep in mind that conventionally, image classification techniques are much more expensive, computation-wise. For instance, typical network architectures such as ResNet34 [64] and VGG16 [65] have a total number of parameters that is about 21 M and 138 M, respectively. That being said, compared to conventional methods, our proposed method can extract more information-rich features from the beam energy images, thus achieving a significant improvement in the reduction of the IUD estimation error. Finally, once the network is fully trained, the total number of basic operations (addition and multiplication) to be performed is constant and grows linearly with the number of users. The such number of operations could be justified for the sake of achieving an estimation error that is of the order of a few centimeters.

## 6. Conclusions

In this paper, we proposed a novel approach for IUD estimation using low-resolution beam energy images. The approach relies on the difference between the user-generated beam energy images to estimate the distance between each pair of users. We then applied a super-resolution technique to improve the IUD estimation accuracy with low-resolution beam energy images. Our experiments show that our method can achieve a distance estimation error equal to 0.13 m for a coverage area of 60 × 30 m^2^. Our method outperforms the conventional methods based on user location to measure the IUD. Besides, applying super-resolution to images of resolution 4 × 4 and 8 × 8, improving their resolution to 8 × 8 and 16 × 16, respectively, has led to a further improvement in the estimation of the distance between the users. Compared with the original 4 × 4 and 8 × 8 images, the enhanced versions of these images by super-resolution exhibit better performance in the estimation of IUD. In fact, they achieve an estimation accuracy comparable to the original 8 × 8 and 16 × 16 images, respectively. The propose method still has some limitations. Even though the proposed method is usable even in scenarios where the users are moving, we can only get the outdated beam energy image of users. Thus, the accuracy of detection will be highly affected by the frequency of beam sweeping: if the sweeping is very frequent, a high accuracy can be obtained, however, it will lead to a huge over head. On the other hand, if the sweeping is not very frequent, the overhead is reduced, however, a drop in the accuracy is expected. Identifying a good balance between the accuracy and the sweeping frequency is yet to be identified. Nonetheless, we could use a different type of neural network (ConvLSTM) to account for the change over time of user’s location and predict the beam energy image of the users based on their history [55] to reduce the frequency of the sweeping, while accurately predicting their distances.

## Figures and Tables

**Table 1:** Key Notations.

Symbol	Description
*x*	Scalar.
x	Column-vector.
X	Matrix.
||·||	The $l_{2}$-norm operator.
⊗	Kronecker product.
${\left(\cdot \right)}^{H}$	Complex conjugate transpose.
${\left(\cdot \right)}^{T}$	The transpose operations.
*P*	The transmit power.
$s_{k}$	The transmitted signal of the *k*-th user.
$w_{RF}^{k}$	The analog beamformer of the *k*-th user.
$r_{k}$	The received signal of the *k*-th user.
W	The Discrete Fourier Transform based codebook.
$n_{k}$	Additive white Gaussian noise.
$h_{k}$	The mmWave mMIMO channel between the BS and the *k*-th user.
*L*	The total number of paths.
φ	The horizontal angles.
ϕ	The vertical angles.
$\alpha_{\ell }$	The complex path gain of the *ℓ*-th path.
$N_{t}$	The number of transmit antennas.
$N_{v}$	The number of antennas along the vertical.
$N_{h}$	The number of antennas along the horizontal.
*g*	The complex reflection gain.
$d_{p}$	The path distance.
λ	The wavelength.
*d*	The distance between the consecutive antennas in both vertical and horizontal directions.
$n_{b}^{v}$	The number of beams at the vertical axis.
$n_{b}^{h}$	The number of beams at the horizontal axis.
$w_{k,n}^{v}$	The weights on antenna elements along the vertical directions.
$w_{k,n}^{h}$	The weights on antenna elements along the horizontal directions.
$m_{k}$	The Received Signal Strength Indicator (RSSI) of the received beams.
*e*	The distance estimation error.
M	The generated power matrices/images.
$D_{k,l}$	The difference matrix between the two users *k* and *l*.
$S_{b}$	Batch size.

**Table 3:** The Simulations Parameter Settings.

Parameter Description	Value
Carrier frequency	60 GHz
# Antennas at the BS ($N_{v}\;\times \;N_{h}$)	8 × 8
# Number of beams $N_{b}$	16 × 16/8 × 8/4 × 4
user spread area	60 × 30 m^2^
Height of BS	10 m
Total downlink power *P*	30 dBm
Signal to interference power ratio	10 dB
Number of paths *L*	25
Reflection gain *g*	−6 dB
Noise figure *F*	9.5 dB

**Table 4:** A Summary of the Distance Estimation Error at CDF = 0.5 and at CDF = 0.9.

	CDF = 0.5	CDF = 0.9
DCNN	0.280 m	0.703 m
Classification	0.409 m	1.018 m
Regression	0.780 m	1.304 m
4 × 4	0.160 m	0.304 m
4 × 4 enhanced	0.101 m	0.231 m
8 × 8	0.097 m	0.205 m
8 × 8 enhanced	0.096 m	0.197 m
16 × 16	0.093 m	0.184 m

**Table 5:** A Summary of the Distance Estimation Error at CDF = 0.5 and at CDF = 0.9 on the new environment using different number of users.

	CDF = 0.5	CDF = 0.9
4 × 4 fine-tuned [20 users]	0.873 m	2.828 m
4 × 4 fine-tuned [50 users]	0.284 m	0.919 m
4 × 4 fine-tuned [100 users]	0.266 m	0.862 m
8 × 8 fine-tuned [20 users]	0.710 m	2.307 m
8 × 8 fine-tuned [50 users]	0.153 m	0.497 m
8 × 8 fine-tuned [100 users]	0.142 m	0.461 m

**Table 6:** A Summary of the Distance Estimation Error at CDF = 0.5 and at CDF = 0.9 on the new environment for different epochs.

	CDF = 0.5	CD = 0.9
4 × 4 fine-tuned [10 epochs]	0.873 m	2.826 m
4 × 4 fine-tuned [50 epochs]	0.327 m	1.060 m
4 × 4 fine-tuned [100 epochs]	0.284 m	0.919 m
8 × 8 fine-tuned [10 epochs]	0.655 m	2.129 m
8 × 8 fine-tuned [50 epochs]	0.218 m	0.710 m
8 × 8 fine-tuned [100 epochs]	0.153 m	0.497 m

**Table 7:** Complexity of the proposed method and that of DCNN.

Model	Total Params
4 × 4	1,461,121
4 × 4 enhanced	2,861,537
8 × 8	1,657,729
8 × 8 enhanced	3,058,145
16 × 16	2,444,161
DCNN [32]	41,401
Classification [31]	85,332
Regression [47]	61,231

## Data Availability

Not applicable.

## Abbreviations

The following abbreviations are used in this manuscript:

ADP	Angle Delay Profile
AoA	Angle of Arrival
AWGN	Additive white Gaussian noise
BS	Base Station
CDF	Cumulative Distribution Function
CNN	Convolutional Neural Network
COCOA	COVID-19 Contact Confirming Application
CSI	Channel State Information
D2D	Device-to-Device
DNN	Deep Neural Network
DCNN	Deep Convolutional Neural Network
DFT	Discrete Fourier Transform
DNN	Deep Neural Network
MIMO	Multiple-Input Multiple-Output
mmWave	MillimeterWave
MSE	Mean Squared Error
ReLU	Rectified Linear Unit
RSSI	Received Signal Strength Indicator
ToA	Time of Arrival
UPA	uniform Planer Array
